# Supplementary material for: Epidemiology and molecular characterisation of multidrug-resistant Escherichia coli isolated from chicken meat
Source: PLoS One. 2025 May 14;20(5):e0323909. doi: 10.1371/journal.pone.0323909 (PMC12077676; doi:10.1371/journal.pone.0323909)
Supplement: S1 Table — (DOCX) [file pone.0323909.s001.docx]

Supplementary Table S1: List of sample collected from different LBMs and supermarkets (LBM= Live Bird Market, SM= Supermarket)

| Sources | Category | Sample type | |
| --- | --- | --- | --- |
|  |  | Liver | Muscle |
| LBMs | LBM-1 | 10 | 10 |
|  | LBM-2 | 10 | 10 |
|  | LBM-3 | 10 | 10 |
|  | LBM-4 | 10 | 10 |
|  | LBM-5 | 10 | 10 |
|  | LBM-6 | 10 | 10 |
|  | LBM-7 | 10 | 10 |
|  | LBM-8 | 10 | 10 |
|  | LBM-9 | 10 | 10 |
| Supermarkets | SM-1 | 25 | 25 |
|  | SM-2 | 25 | 25 |
|  | SM-3 | 25 | 25 |
|  | SM-4 | 25 | 25 |
|  | SM-5 | 25 | 25 |
