# Supplementary material for: Epidemiology and molecular characterisation of multidrug-resistant Escherichia coli isolated from chicken meat
Source: PLoS One. 2025 May 14;20(5):e0323909. doi: 10.1371/journal.pone.0323909 (PMC12077676; doi:10.1371/journal.pone.0323909)
Supplement: Attachment — Submitted filename: PONE-D-25-06137 rev AAO.pdf [file pone.0323909.s003.pdf]

PLOS ONE

Epidemiology and Molecular Characterisation of Multidrug-Resistant Escherichia coli Isolated from Chicken Meat

--Manuscript Draft--

Manuscript Number:	PONE-D-25-06137
Article Type:	Research Article
Full Title:	Epidemiology and Molecular Characterisation of Multidrug-Resistant Escherichia coli Isolated from Chicken Meat
Short Title:	Multidrug-Resistant Escherichia coli in Chicken Meat
Corresponding Author:	Mohammad Mahmudul Hassan, PhD<br>CVASU: Chattogram Veterinary and Animal Sciences University<br>Chattogram, CTG BANGLADESH
Keywords:	Chicken meat; E. coli; MDR; genes; Public Health
Abstract:	This study aimed to investigate the prevalence and antimicrobial resistance (AMR) profiles of Escherichia coli (E. coli) in broiler meat samples from live bird markets (LBMs) and supermarkets (SMs) in the Chattogram Metropolitan Area (CMA), Bangladesh. A total of 430 samples, comprising 215 liver and 215 muscle samples, were collected between October 2020 and February 2021 from nine LBMs and five SMs. Samples were processed and cultured, and E. coli was isolated and identified through phenotypic and molecular methods, including PCR targeting the uidA and uspA genes. Antimicrobial susceptibility testing (AST) was performed using the Kirby-Bauer disc diffusion method against seven antibiotics representing six different antimicrobial classes. The study found an overall prevalence of 56.28% (95% CI: 51.56–60.89) for E. coli. The prevalence in LBMs (58.33%) was higher than in SMs (54.80%), with liver samples showing a slightly higher rate of contamination (63.33% in LBMs, 55.20% in SMs) compared to muscle samples. AMR profiling revealed high resistance rates to sulfamethoxazole-trimethoprim (88.84%), tetracycline (86.78%), and ampicillin (82.23%). Conversely, cephalexin (63.64%) and gentamicin (57.02%) had the highest susceptibility rates. A significant proportion (84.71%) of isolates were multidrug-resistant (MDR), with some isolates resistant to up to six classes of antimicrobial. The multiple antibiotic resistance (MAR) index ranged from 0.14 to 1.00, indicating substantial antimicrobial exposure. PCR analysis confirmed the presence of the blaTEM gene in all ampicillin-resistant isolates, while 75.35% of sulfamethoxazole-resistant isolates carried the sul2 gene. Correlation analysis showed a strong association between phenotypic resistance to ampicillin and the blaTEM gene (r = 1), and moderate correlations between sul 2 and sulfamethoxazole resistance (r = 0.5). These findings underscore the widespread occurrence of MDR E. coli in broiler meat, posing a public health risk.
Order of Authors:	Hamida Khanom
	Chandan Nath
	Philip P. Mshelbwala, PhD
	Md Ridoan Pasha
	Ricardo Soares Magalhaes, PhD
	John I. Alawneh, PhD
	Mohammad Mahmudul Hassan, PhD
Additional Information:	
Question	Response
**Financial Disclosure**<br><br>Enter a financial disclosure statement that describes the sources of funding for the	The author(s) received no specific funding for this work.

Powered by Editorial Manager® and ProduXion Manager® from Aries Systems Corporation

work included in this submission. Review the submission guidelines for detailed requirements. View published research articles from *PLOS ONE* for specific examples.<br><br>This statement is required for submission and **will appear in the published article** if the submission is accepted. Please make sure it is accurate.<br><br>**Funded studies**<br>Enter a statement with the following details:<br>• Initials of the authors who received each award<br>• Grant numbers awarded to each author<br>• The full name of each funder<br>• URL of each funder website<br>• Did the sponsors or funders play any role in the study design, data collection and analysis, decision to publish, or preparation of the manuscript?<br><br>Did you receive funding for this work?	
**Competing Interests**<br><br>Use the instructions below to enter a competing interest statement for this submission. On behalf of all authors, disclose any competing interests that could be perceived to bias this work—acknowledging all financial support and any other relevant financial or non-financial competing interests.<br><br>This statement is required for submission and **will appear in the published article** if the submission is accepted. Please make sure it is accurate and that any funding sources listed in your Funding Information later in the submission form are also declared in your Financial Disclosure statement.<br><br>View published research articles from *PLOS ONE* for specific examples.	The authors have declared that no competing interests exist.

**NO authors have competing interests**<br><br>Enter: *The authors have declared that no competing interests exist.*<br><br>**Authors with competing interests**<br><br>Enter competing interest details beginning with this statement:<br><br>*I have read the journal's policy and the authors of this manuscript have the following competing interests: [insert competing interests here]*<br><br>* typeset	
**Ethics Statement**<br><br>Enter an ethics statement for this submission. This statement is required if the study involved:<br><br>• Human participants<br>• Human specimens or tissue<br>• Vertebrate animals or cephalopods<br>• Vertebrate embryos or tissues<br>• Field research<br><br>Write "N/A" if the submission does not require an ethics statement.<br><br>General guidance is provided below. Consult the submission guidelines for detailed instructions. **Make sure that all information entered here is included in the Methods section of the manuscript.**	This study was conducted by following the Declaration of Helsinki, and the protocol was approved by the Ethics Committee of the Chattogram Veterinary and Animal Sciences University, Bangladesh (permit reference number: CVASU/Dir (R and E) EC/2019/126 (02), Date: 29 December 2019).

Powered by Editorial Manager® and ProduXion Manager® from Aries Systems Corporation

**Format for specific study types**<br><br>**Human Subject Research (involving human participants and/or tissue)**<br>• Give the name of the institutional review board or ethics committee that approved the study<br>• Include the approval number and/or a statement indicating approval of this research<br>• Indicate the form of consent obtained (written/oral) or the reason that consent was not obtained (e.g. the data were analyzed anonymously)<br><br>**Animal Research (involving vertebrate animals, embryos or tissues)**<br>• Provide the name of the Institutional Animal Care and Use Committee (IACUC) or other relevant ethics board that reviewed the study protocol, and indicate whether they approved this research or granted a formal waiver of ethical approval<br>• Include an approval number if one was obtained<br>• If the study involved *non-human primates*, add *additional details* about animal welfare and steps taken to ameliorate suffering<br>• If anesthesia, euthanasia, or any kind of animal sacrifice is part of the study, include briefly which substances and/or methods were applied<br><br>**Field Research**<br><br>Include the following details if this study involves the collection of plant, animal, or other materials from a natural setting:<br>• Field permit number<br>• Name of the institution or relevant body that granted permission	
**Data Availability**<br><br>Authors are required to make all data underlying the findings described fully available, without restriction, and from the time of publication. PLOS allows rare exceptions to address legal and ethical concerns. See the PLOS Data Policy and FAQ for detailed information.	Yes - all data are fully available without restriction

Powered by Editorial Manager® and ProduXion Manager® from Aries Systems Corporation

A Data Availability Statement describing where the data can be found is required at submission. Your answers to this question constitute the Data Availability Statement and **will be published in the article**, if accepted.<br><br>**Important:** Stating 'data available on request from the author' is not sufficient. If your data are only available upon request, select 'No' for the first question and explain your exceptional situation in the text box.<br><br>Do the authors confirm that all data underlying the findings described in their manuscript are fully available without restriction?	
**Describe where the data may be found in full sentences. If you are copying our sample text, replace any instances of XXX with the appropriate details.**<br><br>• If the data are **held or will be held in a public repository**, include URLs, accession numbers or DOIs. If this information will only be available after acceptance, indicate this by ticking the box below. For example: *All XXX files are available from the XXX database (accession number(s) XXX, XXX.).*<br>• If the data are all contained **within the manuscript and/or Supporting Information files**, enter the following: *All relevant data are within the manuscript and its Supporting Information files.*<br>• If neither of these applies but you are able to provide **details of access elsewhere**, with or without limitations, please do so. For example:<br><br>*Data cannot be shared publicly because of [XXX]. Data are available from the XXX Institutional Data Access / Ethics Committee (contact via XXX) for researchers who meet the criteria for access to confidential data.*<br><br>*The data underlying the results presented in the study are available from (include the name of the third party*	All relevant data are within the manuscript and its Supporting Information files.

and contact information or URL).<br>• This text is appropriate if the data are owned by a third party and authors do not have permission to share the data.<br><br>* typeset	
Additional data availability information:	

Main_Manuscript Click here to access/download;Manuscript;Main_Manuscript.docx

Epidemiology and Molecular Characterisation of Multidrug-Resistant Escherichia coli Isolated from Chicken Meat

Hamida Khanom[1,2] Chandan Nath[1], Philip P. Mshelbwala[3], Md Ridoan Pasha[1], Ricardo Soares Magalhaes[4,5], John I. Alawneh[6], Mohammad Mahmudul Hassan[1, 4]*

[1]Faculty of Veterinary Medicine, Chattogram Veterinary and Animal Sciences University, Chattogram 4225, Bangladesh; hamidakhanom21@gmail.com; chandannath227@gmail.com; pasha.dpbp@cvasu.ac.bd; miladhasan@cvasu.ac.bd

[2]Department of Biosciences and Biotechnology, Fukui Prefectural University, Yoshida-gun 910-1195, Fukui, Japan

[3]Department of Primary Industries, Orange, NSW 2800, Australia; philip.mshelbwala@dpi.nsw.gov.au

[4]Queensland Alliance for One Health Sciences, School of Veterinary Science, The University of Queensland, Gatton, QLD 4343, Australia; r.magalhaes@uq.edu.au

[5]Children's Health and Environment Program, Children Health Research Centre, The University of Queens-land, Brisbane, Queensland, Australia

[6]Plant Biosecurity and Product Integrity, Biosecurity Queensland, Department of Agriculture and Fisheries, Brisbane, QLD 4000, Australia; john.alawneh@daf.qld.gov.au

***Correspondence**: Mohammad Mahmudul Hassan; miladhasan@cvasu.ac.bd

Abstract

Ensuring the safety of poultry products is critical for public health, particularly due to the rising concern of antimicrobial resistance (AMR) in foodborne pathogens. This study aimed to investigate the prevalence and antimicrobial resistance (AMR) patterns of *Escherichia coli (E. coli)* isolated from broiler chicken meat samples collected from live bird markets (LBMs) and supermarkets (SMs) in the Chattogram Metropolitan Area (CMA), Bangladesh. A total of 430 samples, comprising 215 liver and 215 muscle samples, were collected between October 2020 and February 2021 from nine LBMs and five SMs. Samples were processed and cultured, and *E. coli* was isolated and identified through phenotypic and molecular techniques, including PCR targeting the *uid*A and *usp*A genes. Antimicrobial susceptibility testing (AST) was conducted using the Kirby-Bauer disk diffusion techniques with seven antibiotics from six distinct antimicrobial classes. The study found an overall prevalence of 56.28% (95% CI: 51.56–60.89) for *E. coli*. The prevalence in LBMs (58.33%) was higher than in SMs (54.80%), with liver samples showing a slightly higher rate of contamination (63.33% in LBMs, 55.20% in SMs) compared to muscle samples. AMR profiling revealed high resistance rates to sulfamethoxazole-trimethoprim (88.84%), tetracycline (86.78%), and ampicillin (82.23%). Conversely, cephalexin (63.64%) and gentamicin (57.02%) had the highest susceptibility rates. A significant proportion (84.71%) of isolates were multidrug-resistant (MDR), with some isolates resistant to up to six classes of antimicrobial. The multiple antibiotic resistance (MAR) index ranged from 0.14 to 1.00, indicating substantial

antimicrobial exposure. PCR analysis confirmed the presence of the *bla*TEM gene in all ampicillin-resistant isolates, while 75.35% of sulfamethoxazole-resistant isolates carried the *sul*2 gene. Correlation analysis revealed a strong association between phenotypic resistance to ampicillin and the presence of the *bla*TEM gene (r = 1), along with a moderate correlation between *sul2* and resistance to sulfamethoxazole (r = 0.5). These findings highlight the widespread presence of multidrug-resistant (*MDR*) *E. coli* in broiler meat, posing a significant public health concern.

Keywords: Chicken meat, *E. coli*, MDR, genes, public health

1. Introduction

Escherichia coli (E. coli) is a bacterium essential for supporting intestinal health in both humans and animals [1]. Although the majority of *E. coli* strains are non-pathogenic, approximately 10–15% of intestinal coliforms comprise opportunistic and pathogenic serotypes capable of causing infections in immunocompromised hosts, including poultry [2]. Contamination of meat with *E. coli* is frequently associated with poor slaughter hygiene [3], and the strains isolated from such contaminated meat have shown resistance to commonly used antibiotics [4]. This antimicrobial resistance presents serious threats to the health of both humans and animals [5]. While the majority of *E. coli* strains coexist harmlessly in the large intestine, they can become pathogenic under specific conditions, causing both intestinal and systemic infections [6]. Antimicrobials are extensively used in both human and veterinary medicine to manage infections and health risks, but their overuse contributes to the rise of AMR, posing a serious challenge to effective treatment and public health [7]. Antimicrobials are often administered as growth promoters (AMGP) in poultry farming, especially for broilers. However, to minimise the risk of resistance development, they should be used cautiously and primarily for therapeutic and preventive purposes [8]. Overuse of antibiotics accelerates the development of antibiotic-resistant bacteria, a complex process driven by bacterial genetic and metabolic mechanisms [9; 10]. In intensive broiler chicken production, the strong antibiotic selection pressure has led to a substantial presence of resistant bacteria in poultry fecal flora, which can then spread to humans, pets, and the environment [11-13]. The routine use of antimicrobials in livestock production contributes to developing AMR bacteria, which can be spread to humans via the food chain, particularly through broiler chicken meat, posing serious health risks [14].

Poor handling, inadequate cleaning, and improper meat-selling practices are known to contribute to poultry meat contamination with *E. coli* [15]. Poultry meat, particularly broiler chicken meat, has been recognised as a likely source of infection due to the high risk of contamination during meat processing [16]. While the global prevalence of avian *E. coli* in broiler meat has been well documented, there is still limited information on the situation in Bangladesh. Despite the presence of veterinary and research laboratories across Bangladesh, systematic surveillance of AMR remains inadequate. Over 60% of farmers in Bangladesh reportedly use antibiotics without prescriptions [17]. Chicken meat from LBMs has AMR, as vendors often use antibiotics to reduce mortality rates among their stock [18]. Moreover, a range of antimicrobials, including ciprofloxacin, amoxicillin, sulfamethoxazole-trimethoprim, and gentamicin, are frequently used at various stages of poultry production, contributing to the development of antibiotic-resistant *E. coli* strains in Bangladesh. This study examines the

current levels of *E. coli* contamination and AMR profiles, including *bla*TEM, *sul*1, and *sul*2 genes, in broiler chicken meat from SMs and LBMs in the CMA of Bangladesh.

2. Methodology

2.1 Study location and design

This study was conducted between October 2020 and February 2021 in the Chattogram Metropolitan Area (CMA) of the Chattogram district of Bangladesh. This research is a part of a larger project, with some components already published [18; 19]. Nine LBMs and five SMs were conveniently selected for sample collection within the CMA. Ten stalls were randomly selected from each LBM. The geographical locations of the sampling areas are shown in

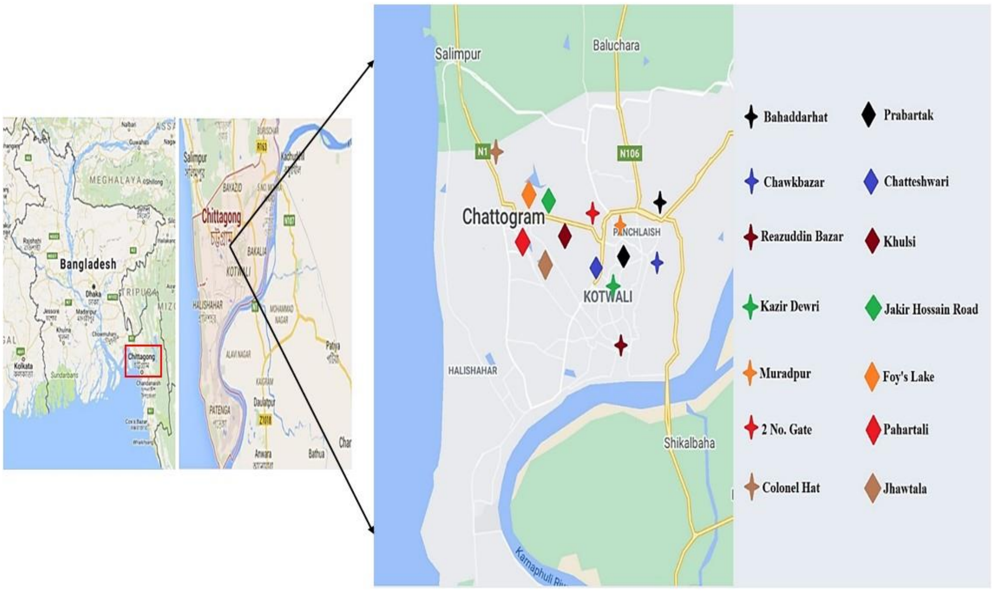

Figure 1.

Figure 1: Map depicts locations of selected LBMs and SMs of Chattogram Metropolitan Area

2.2 Sample collection, transportation, and processing

From each LBM, 10 liver samples and 10 breast muscle samples were collected; from each SM, 25 liver samples and 25 muscle samples were collected. In total, 430 samples were obtained, consisting of 215 liver and 215 muscle samples. An overview of the sample collection is presented in Supplementary Table S1. The samples were collected in separate zipper bags while maintaining proper hygiene procedures. After collection, they were transported to the Department of Microbiology and Veterinary Public Health (DMVPH) at Chattogram Veterinary and Animal Sciences University (CVASU) for further investigation, ensuring the cold chain was maintained throughout transport. The samples were then processed into small pieces using sterile scissors, and 1 g of each sample was transferred into a separate sterile Falcon tube containing 9 mL of buffered peptone water (BPW) (HIMEDIA, pH: 7.0±0.2, Mumbai, India). The samples were incubated at 37°C overnight for primary enrichment.

2.3 Isolation and presumptive detection of *E. coli*

To isolate *E. coli*, the enriched culture was streaked onto MacConkey agar medium (HIMEDIA, pH: 7.1±0.2, Mumbai, India) and incubated at 37°C for 24 hours. Bright pink-coloured, large colonies on the MacConkey agar plate were suspected of *E. coli*. These colonies were then streaked onto Eosin Methylene Blue (EMB) agar plates (HIMEDIA, pH: 7.0±0.2, Mumbai, India) and incubated at 37°C for 24 hours. A "green metallic sheen" on the EMB agar confirmed the growth of *E. coli*. Following confirmation, the isolates were inoculated onto blood agar (HIMEDIA, pH: 7.0±0.2, Mumbai, India) and incubated at 37°C for 24 hours. All confirmed *E. coli* isolates were cultured in brain heart infusion (BHI) broth (HIMEDIA, pH: 7.0±0.2, Mumbai, India) and incubated overnight at 37°C. For each isolate, 700 µL of the BHI broth culture was mixed with 300 µL of 15% glycerol in an Eppendorf tube. The tubes were properly labelled and stored at -80°C for further investigation.

2.4 Molecular detection of *E. coli*

Phenotypic *E. coli* isolates were subjected to molecular identification by PCR. Genomic DNA was extracted using the crude boiling method described by Malorny et al. [20]. Molecular identification of *E. coli* was carried out through species-specific multiplex PCR in a thermal cycler (DLAB, USA) using primers targeting the *uid*A gene and the flanking region of the *usp*A gene [21]. The oligonucleotide primer sequences are listed in Table 1. The PCR reaction mixture consisted of 12.5 µL of OneTaq Quick-load 2X Master Mix with Standard Buffer (New England Biolabs Inc.), 0.5 µL each of the forward and reverse primers (10 pmol), 1 µL of template DNA, and the required volume of nuclease-free water. The thermal cycling conditions were as follows: initial denaturation at 94°C for 5 minutes, followed by 35 cycles of denaturation at 94°C for 10 seconds, annealing at 55.2°C for 10 seconds, extension at 72°C for 1 minute, and a final extension at 72°C for 10 minutes. After amplification, 5 µL of the PCR products were loaded onto a 1.5% (w/v) agarose gel, prepared using agarose powder (MP Biomedicals, USA) and 1X TAE buffer (Thermo Fisher Scientific, USA). The gel was visualised in a gel documentation system (UVP UVsolo touch, Analytik Jena AG, Thermo Fisher Scientific, USA) after staining with ethidium bromide (Sigma-Aldrich, USA).

2.5 AST of *E. coli*

The *E. coli* positive isolates in PCR were screened for antimicrobial susceptibility against a panel of antimicrobials using the Kirby-Bauer disc diffusion method [22]. Seven antimicrobials of six different groups such as penicillins: ampicillin, tetracyclines: tetracycline and doxycycline, aminoglycosides: gentamicin, fluoroquinolones: ciprofloxacin, sulfonamides: sulfamethoxazole-trimethoprim, and cephalosporins: cephalexin) of drugs that had public health significance were selected for antimicrobial susceptibility testing. The following antimicrobials with respective disc potencies were used: TE: tetracycline (30µg), CN: gentamicin (10µg), DO: doxycycline (30µg), AMP: ampicillin (10µg), CL: cephalexin (30µg), SXT: sulfamethoxazole-trimethoprim (23.75µg +1.25 µg), CIP: ciprofloxacin (5µg). After dispensing all the discs, the Mueller Hinton agar (HIMEDIA, pH: 7.0±0.2, Mumbai, India) plates were incubated at 37°C for 18 hours. After incubation, the size of the zone of inhibition (in mm) around a disc, including the diameter of the disc, was measured using slide calipers, and the result was interpreted according to Clinical Laboratory Standards Institute guidelines [23]. The pan-susceptible *E. coli* ATCC 25922 was used as a quality control strain during AST. The *E. coli* isolates resistant to at least three antimicrobial classes were described as multidrug-resistant (MDR) [24]. The multiple antibiotic resistance (MAR) index was

estimated by following the formula a/b, a= the number of antibiotics that showed resistance by isolates, b= the number of antibiotics that were exposed by isolates, as described earlier by Krumperman et al., [25].

2.6 Detection of antimicrobial resistance genes

Using PCR, the phenotypic sulphonamide- and ampicillin-resistant isolates were tested for the presence of resistance genes. The *sul* 1 and *sul* 2 genes were screened for sulphonamide resistance, and the *bla*TEM gene for ampicillin resistance, as previously described by Lanz et al. [26] and Hasman et al. [27]. The oligonucleotide primer sequences, amplicon sizes, and annealing temperatures are provided in Table 1.

Table 1: The oligonucleotide primer sequences used in this study

Target gene	Primer Name	Primer sequence (5′-3′)	Annealing temperature	Amplicon size (bp)	References
*usp*A	*usp*A -F	CCGATACGCTGCCAATCAGT	55.2 °C	884	[21]
	*usp*A -R	ACGCAGACCGTAGGCCAGAT			
*uid*A	*uid*A-F	TATGGAATTTCGCCGATTTT		164	
	*uid*A -R	TGTTTGCCTCCCTGCTGCGG			
bla_{TEM}	bla_{TEM} -F	GCGGAACCCCTATTTG	50 °C	964	[27]
	bla_{TEM} -R	TCTAAAGTATATATGAGTAAACTTGGTCTGAC			
sul 1	*sul* 1- F	GTGACGGTGTTCGGCATTCT	68 °C	779	[26]
	sul 1- R	TCCGAGAAGGTGATTGCGCT			
sul 2	*sul* 2- F	CGGCATCGTCAACATAACCT	66 °C	721	
	sul 2- R	TGTGCGGATGAAGTCAGCTC			

2.7 Statistical analysis

All sampling and laboratory test data were meticulously entered, organised, and cleaned using Microsoft Excel 2019. Descriptive statistics, including percentages and 95% confidence intervals, were performed using the modified Wald method available in the GraphPad QuickCalcs online tool (https://www.graphpad.com/quickcalcs/). Data visualisations, such as heat maps and bar charts, were generated using GraphPad Prism 7.0 (GraphPad Software, La Jolla, CA, USA) to illustrate trends and distributions effectively. Correlation analyses between antimicrobial coefficients and corresponding resistance gene abundances were conducted using R software (version 4.4.1; https://www.r-project.org/). These correlations were visualised with high-quality graphical outputs produced in R, ensuring precise and informative data presentation.

3. Results

3.1 Prevalence of *E. coli* in LBMs and SMs

Among the 450 samples tested in this study, 242 (56.28%, 95% CI: 51.56–60.89) were confirmed as *E. coli*. In LBMs, the prevalence of *E. coli* was 58.33%, with 63.33% in liver and 52.22% in muscle samples. Similarly, the prevalence in SMs was 54.80%, with 55.20% in liver samples and 54.40% in muscle samples. The prevalence of *E. coli* across different sources is presented in Table 2.

Table 2: The overview of the sample collected and frequency of *E. coli* from different sources

Sources	Samples	N, *E. coli*	Percentage (%), (95% CI)
LBMs	Liver (n=90)	57	63.33 (53.01-72.57)
	Muscle (n=90)	48	52.22 (42.02-62.24)
subtotal (n= 180)		105	58.33 (51.03-65.29)
SMs	Liver (n=125)	69	55.20 (46.46-63.63)
	Muscle (n=125)	68	54.40 (45.67- 62.87)
subtotal (n= 250)		137	54.80 (48.60-60.85)
Total (N= 430)		242	56.28 (51.56-60.89)

3.2 AMR patterns of *E. coli* isolates of LBMs and SMs

The AST showed that the highest proportion of isolates were resistant to sulfamethoxazole-trimethoprim (88.84%), followed by tetracycline (86.78%) and ampicillin (82.23%). In contrast, the highest susceptibility was confirmed for cephalexin (63.64%), followed by gentamicin (57.02%) and ciprofloxacin (23.14%). Additionally, 28.51% of the isolates were intermediate to doxycycline. The AMR profiles are illustrated in Figure 2/a.

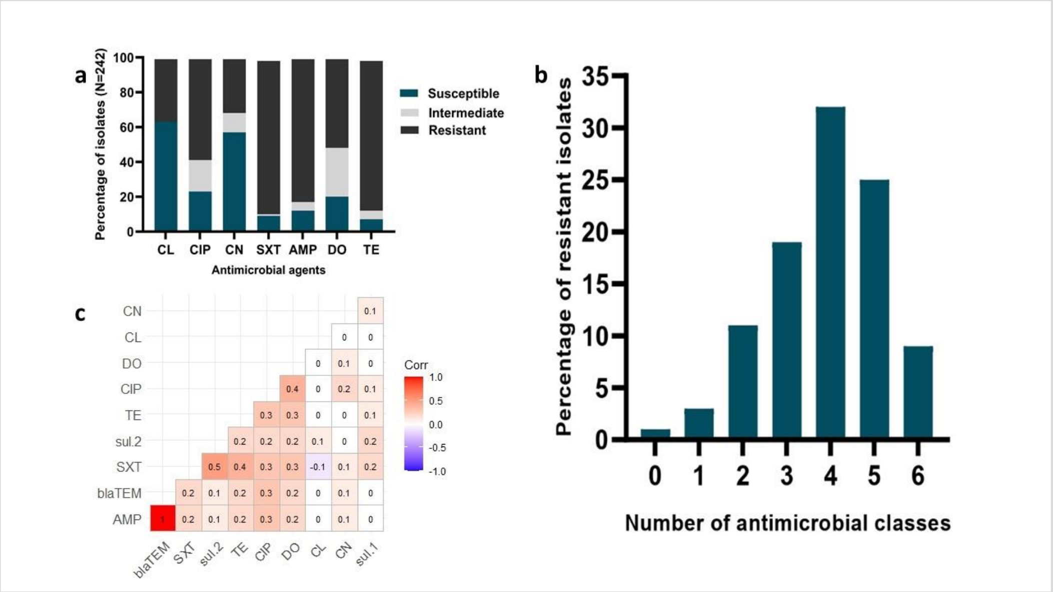

Figure 2: AMR patterns of *E. coli* isolated from broiler chicken muscle and liver samples. a) AMR patterns, b) MDR patterns and c) correlation coefficient of specific antimicrobials and

resistance genes. Here, TE= tetracycline, DO= doxycycline CN= gentamicin, CIP= ciprofloxacin, SXT= sulfamethoxazole-trimethoprim, CL= cephalexin and AMP= ampicillin.

3.3 MDR profiles of *E. coli*

This study revealed that 205 isolates (84.71%) exhibited multidrug resistance (MDR), while 37 isolates (15.29%) were not MDR. Among the non-MDR isolates, 3 (1.24%) showed no resistance to any antimicrobials tested. Of the MDR isolates, 78 (32.23%) were resistant to four antimicrobial classes (Figure 2/b). The phenotypic antimicrobial resistance patterns revealed that 32 isolates (13.22%) were resistant to four antimicrobial classes, displaying the CIP+SXT+AMP+DO+TE resistance patterns. Additionally, 25 isolates (10.33%) exhibited resistance to five antimicrobial classes, displaying the CIP+CN+SXT+AMP+DO+TE resistance pattern. Furthermore, 15 isolates (6.22%) demonstrated resistance to six antimicrobial classes following the CL+CIP+CN+SXT+AMP+DO+TE resistance pattern (Table 3). The multiple antibiotic resistance (MAR) index for all isolates ranged from 0.14 to 1 (Table 3).

Table 3: The phenotypic AMR patterns of *E. coli* isolates, along with the MAR index

No of isolates	%	Resistance type	Phenotypic resistance patterns	MAR Index
32	13.22	MDR	Four classes<br>CIP, SXT, AMP, DO, TE	0.57
25	10.33	MDR	Five classes<br>CIP, CN, SXT, AMP, DO, TE	0.86
18	7.44	MDR	Five classes<br>CL, CIP, SXT, AMP, DO, TE	0.86
18	7.44	MDR	Three classes<br>SXT, AMP, TE	0.43
15	6.20	MDR	Six classes<br>CL, CIP, CN, SXT, AMP, DO, TE	1
12	4.96	MDR	Four classes<br>CIP, SXT, AMP, TE	0.57
8	3.31	MDR	Three classes<br>SXT, AMP, DO, TE	0.57
7	2.89	MDR	Four classes<br>CL, SXT, AMP, TE	0.57
7	2.89	MDR	Five classes<br>CIP, CN, SXT, AMP, TE	0.71
6	2.48	MDR	Five classes<br>CL, CIP, SXT, AMP, TE	0.71
6	2.48	MDR	Six classes<br>CL, CIP, CN, SXT, AMP, DO	0.86
5	2.07	MDR	Four classes	0.57

			CL, SXT, AMP, DO, TE	
4	1.65	MDR	Four classes<br>CN, SXT, AMP, TE	0.57
3	1.24	MDR	Four classes<br>SXT, AMP, CIP, TE	0.57
3	1.24	MDR	Three classes<br>CL, AMP, TE	0.43
3	1.24	MDR	Three classes<br>CL, SXT, TE	0.43
2	0.83	MDR	Five classes<br>CL, CN, SXT, AMP, DO, TE	0.86
2	0.83	MDR	Five classes<br>CL, CN, SXT, AMP, TE	0.71
2	0.83	MDR	Four classes<br>CL, CIP, AMP, TE	0.57
2	0.83	MDR	Four classes<br>CL, CN, SXT, TE	0.57
2	0.83	MDR	Four classes<br>CIP, CN, SXT, AMP	0.57
2	0.83	MDR	Three classes<br>CIP, SXT, TE	0.43
2	0.83	MDR	Three classes<br>CIP, SXT, DO, TE	0.57
1	0.41	MDR	Three classes<br>CL, AMP, TE	0.43
1	0.41	MDR	Three classes<br>CL, SXT, AMP	0.43
1	0.41	MDR	Three classes<br>CL, CIP, AMP	0.43
1	0.41	MDR	Three classes<br>CL, SXT, DO, TE	0.57
1	0.41	MDR	Three classes<br>CIP, CN, SXT	0.43
1	0.41	MDR	Three classes<br>SXT, AMP, DO	0.43
1	0.41	MDR	Three classes<br>CL, SXT, DO	0.43
1	0.41	MDR	Three classes<br>CL, CIP, TE	0.43
1	0.41	MDR	Three classes<br>CN, SXT, AMP	0.43
1	0.41	MDR	Three classes<br>CIP, CN, SXT, TE	0.57
1	0.41	MDR	Four classes<br>CL, CN, SXT, AMP	0.57
1	0.41	MDR	Four classes<br>CIP, SXT, AMP, DO	0.57
1	0.41	MDR	Four classes	0.57

			CL, CIP, SXT, TE	
1	0.41	MDR	Four classes<br>CL, CN, SXT, DO	0.57
1	0.41	MDR	Four classes<br>CL, CIP, SXT, DO, TE	0.71
1	0.41	MDR	Four classes<br>CN, SXT, AMP, DO, TE	0.71
1	0.41	MDR	Four classes<br>CL, CN, SXT, DO, TE	0.71

3.4 Correlation of Antimicrobials, resistance genes with *E. coli* isolates from LBMs and SMs

Correlation coefficient analysis revealed a strong positive correlation between AMP resistance and the *bla*TEM gene (r = 1), a moderate correlation between SXT resistance and the *sul*2 gene (r = 0.5), and a weak correlation between SXT resistance and the *sul1* gene (r = 0.2). The weak correlation (r = 0.2) between *bla*TEM and SXT, TE, or *sul*2 indicates an indirect or minimal relationship (Figure 2/c). The heat map compares the AMR profiles of liver and meat samples collected from LBMs and SMs alongside the *sul*1, *sul*2, and *bla*TEM genes associated with sulfonamide and ampicillin resistance (Figure 3).

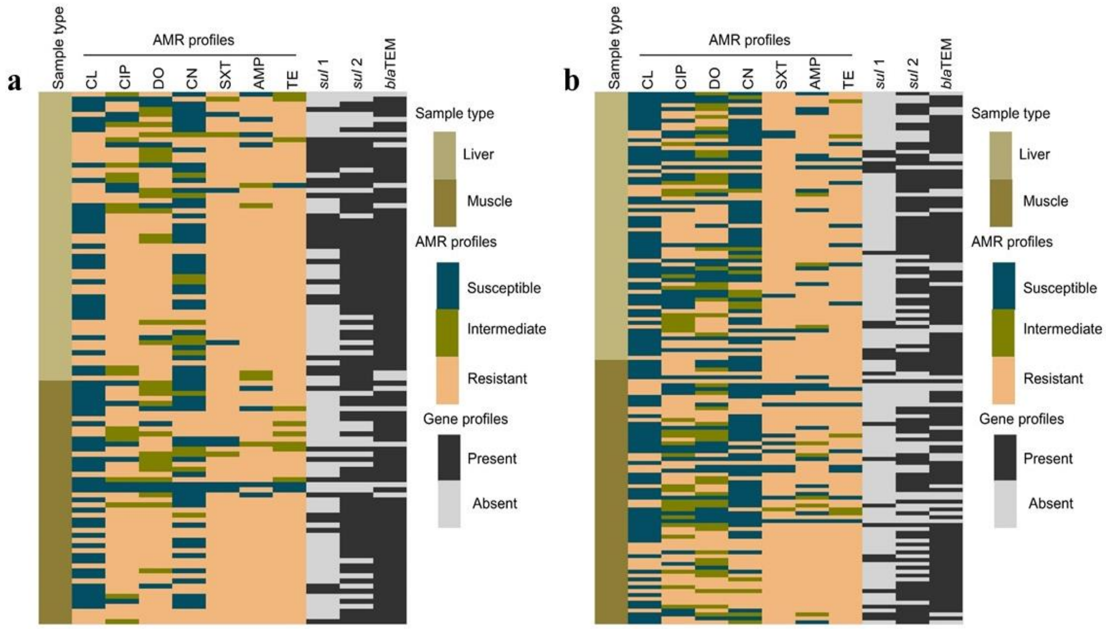

Figure 3: The heatmap illustrates the AMR profiles of liver and muscle samples, along with resistance genes, a) LBMs, b) SMs

4. Discussion

AMR is an escalating global issue as the effectiveness of antimicrobials continues to decline. The results of this study demonstrated a high prevalence of *E. coli* in broiler breast muscle and liver samples from both SMs and LBMs, with these isolates showing resistance to multiple antimicrobials.

In this study, the prevalence of *E. coli* in broilers from LBMs is consistent with the findings of Hossain et al. [28], who reported a prevalence of 63.6%; Jakaria et al. [29], who found 82%, and Bashar et al. [30], who reported 100% in poultry. The ampicillin resistance observed in this study is consistent with the findings of Islam et al. [31], who reported a 100% resistance rate to penicillin in *E. coli* isolates from poultry. Schroeder et al. [32] documented lower % resistance rates of 49% for ampicillin and 84% for sulfamethoxazole-trimethoprim. In contrast, Parvin et al. [33] reported significantly higher resistance rates, including 89.5% for ampicillin, 88.4% for sulfamethoxazole-trimethoprim, and 84.9% for tetracycline, which closely aligns with the findings of the present study. The correlation analysis revealed a strong association between phenotypic resistance to ampicillin and the presence of the *bla*TEM gene, indicating that *bla*TEM is a key genetic determinant of ampicillin resistance. This result is consistent with findings in similar studies, where *bla*TEM has been identified as a major contributor to β-lactam antibiotic resistance through the production of TEM-type β-lactamases, which hydrolyse ampicillin effectively [34]. The weak correlation (r = 0.2) between *bla*TEM and sul1 or sul2 suggests a limited direct association. However, these genes may be present on mobile genetic elements like plasmids or integrons, which promote co-selection under antibiotic pressure. This could account for the observed weak statistical correlation. The low correlations between *bla*TEM and non-AMP antibiotics indicate that *bla*TEM primarily confers AMP resistance. Nevertheless, indirect associations might occur due to co-selection or co-localization with other resistance genes on shared genetic elements. The moderate correlation of *sul*2 with resistance to sulfamethoxazole, a component of SXT, underscores its role in sulfonamide resistance. However, as SXT resistance involves both sulfonamide (e.g., *sul*2) and trimethoprim resistance genes (e.g., *dfr*A), this partial correlation reflects the multifactorial nature of resistance SXT. The AST showed the highest resistance to the combination of sulfamethoxazole-trimethoprim, followed by tetracycline and ampicillin. Li et al. [35] found that 70.9% of isolates were MDR, while only 6.5% exhibited no resistance to the tested antimicrobials. The detection of MDR *E. coli* in this study is concerning and aligns with the findings of Hassan et al. [28], who reported that 100% of poultry samples tested positive for MDR *E. coli*. The rise of MDR pathogens presents a major threat, as these bacteria have the potential to evolve into 'superbugs,' resulting in treatment failures in poultry and significant public health risks. The widespread occurrence of MDR in poultry is likely due to the overuse of various antibiotic classes, creating intense selection pressure.

Several studies have highlighted poultry farms and their environments, such as litter and wastewater, as major sources of antibiotic residues [28; 36]. Moreover, vegetables and animal products from wet markets and shops have been identified as reservoirs of these residues, further contributing to AMR [37; 38]. Regular exposure to antibiotic residues accelerates the spread of AMR, particularly in developing countries like Bangladesh. Horizontal transmission of resistant bacteria and genes has also been observed on farms due to antimicrobial residues [39; 40]. The improper use of antimicrobials in both human and veterinary medicine plays a significant role in the development of AMR [41]. Lack of knowledge about proper antimicrobial use and the indiscriminate administration of antibiotics are common issues in Bangladesh [42]. Drug sellers and medical representatives often encourage the random use of antimicrobials without prescriptions [42; 43]. Inappropriate dosing exposes bacteria to subtherapeutic levels of antimicrobials, enabling them to develop resistance [44]. High doses, conversely, can lead to tissue residues, further promoting

resistance [45]. In poultry farming, antimicrobials are frequently administered to entire flocks for infection control and growth promotion [46]. While this reduces mortality and increases profitability, it has serious public health consequences. When humans consume poultry meat containing antibiotic residues, they can inadvertently acquire these residues, contributing to the spread of resistant genes, which are transferred horizontally and vertically among bacterial populations [47]. Although pathogenic bacteria are generally absent in the muscle tissues of healthy birds [48], contamination during slaughter and meat processing can introduce bacteria from the gastrointestinal tract, some of which may already be resistant to antimicrobials due to continuous low-dose exposure.

AMR represents a major threat to both veterinary and public health. In response, the World Health Organization (WHO) has classified antimicrobials into three categories—Access, Watch, and Reserve—based on their significance and the risk of resistance [49]. The Access group is recommended for general use, while the Watch group is reserved for cases where Access group options are ineffective. The Reserve group is intended for use when other options are no longer effective [50]. Addressing AMR requires scientific knowledge and evidence-based practices. Rational use of antimicrobials can improve agriculture while simultaneously reducing the problem of AMR, enhancing both profitability and public health [51; 52].

5. Limitations

This study is part of a larger project and focused on broiler meat samples collected from LBMs and SMs in the CMA, which may not fully reflect other regions or retail systems in Bangladesh, potentially limiting the generalisability of the findings. The AST was conducted using the Kirby-Bauer disc diffusion techniques, which, while widely used, provides qualitative rather than quantitative resistance data, potentially limiting the depth of resistance profiling. The study only investigated resistance to seven antibiotics, leaving the resistance profiles against other clinically relevant antimicrobials unexplored. Molecular characterisation in this study was restricted to detecting the *bla*TEM and *sul*2 genes, while other resistance mechanisms, such as efflux pumps or additional resistance genes, were not examined.

6. Conclusion

This study emphasises the high prevalence of *E. coli* contamination and AMR in broiler meat samples from LBMs and SMs in the CMA, Bangladesh. The findings revealed significant resistance to commonly used antibiotics, including sulfamethoxazole-trimethoprim, tetracycline, and ampicillin, with a substantial proportion of isolates exhibiting MDR. The identification of resistance-associated genes, such as *bla*TEM and *sul*2, further highlights the genetic foundation of AMR in these isolates. These results indicate a pressing need for stricter monitoring of antibiotic use in poultry production and robust food safety measures to mitigate public health risks. Addressing AMR must include integrated strategies involving policymakers, the poultry industry, and public health stakeholders.

Acknowledgement

The author sincerely thanks the office staff and lab assistants from the Department of Physiology, Biochemistry, and Pharmacology, as well as the Department of Microbiology and Public Health at Chattogram Veterinary and Animal Sciences University, Chattogram,

Bangladesh. The author also appreciates the support of LBM and SM owners for providing the important information and meat samples.

Ethical Approval

This study was conducted by following the Declaration of Helsinki, and the protocol was approved by the Ethics Committee of the Chattogram Veterinary and Animal Sciences University, Bangladesh (permit reference number: CVASU/Dir (R and E) EC/2019/126 (02), Date: 29 December 2019).

Authors Contribution

Conceptualisation, M.M.H., and H.K.; methodology, M.M.H., H.K, and C.N.; formal analysis, M.M.H., and C.N.; writing—original draft preparation, H.K., M.M.H. and C.N.; writing—review and editing, P.P.M., M.R.P., C.N., R.S.M., J.I.A., and M.M.H.; All authors have read and agreed to the published version of the manuscript.

Conflict of Interest: None to declare.

Funding

Bangladesh Bureau of Education Information and Statistics (BANBEIS), Ministry of Education, People's Republic of Bangladesh, funded this research with project number #SD-2019967.

Data Availability Statement

The data presented in this study are available in the article and supplementary material.

Reference

1. Levine, M.M. 1987. Escherichia coli that cause diarrhea: enterotoxigenic, enteropathogenic, enteroinvasive, enterohemorrhagic, and enteroadherent. J. infect Dis. 1987, 155(3), 377-389.

2. Akond, M.A.; Alam, S.; Hassan, S.M.R; Shirin, M. Antibiotic resistance of Escherichia coli isolated from poultry and poultry environment of Bangladesh. Internet J. Food Saf. 2009, 11, 19-23.

3. Laury, A.; Echeverry, A.; Brashears, M. Fate of Escherichia coli O157: H7 in Meat. Safety of meat and processed meat 2009, 31-53.

4. Mølbak, K. Spread of resistant bacteria and resistance genes from animals to humans–the public health consequences. J. Vet. Med. B 2004 51(8-9), 364-369.

5. Salehi, T.Z.; Bonab, S.F. Antibiotics Susceptibility Pattern of Escherichia coli Strains Isolated from Chickens with Colisepticemia in Tabriz Province, Iran. Int. J. Poult. Sci. 2006, 5, 677–684.

6. Kittana, H.; Gomes-Neto, J.C.; Heck, K.; Geis, A.L.; Segura Muñoz, R.R.; Cody, L.A.; Schmaltz, R.J.; Bindels, L.B.; Sinha, R.; Hostetter, J.M.; Benson, A.K. Commensal

Escherichia coli Strains Can Promote Intestinal Inflammation via Differential Interleukin-6 Production. Front. Immunol. 2018, 9, 2318.

7. Moulin, G.; Cavalié, P.; Pellanne, I.; Chevance, A.; Laval, A.; Millemann, Y.; Colin, P.; Chauvin, C. A Comparison of Antimicrobial Usage in Human and Veterinary Medicine in France from 1999 to 2005. J. Antimicrob. Chemother. 2008, 62, 617–625.

8. Hassan, M.M.; Kalam, M.A.; Alim, M.A.; Shano, S.; Nayem, M.R.K.; Badsha, M.R.; Al Mamun, M.A.; Hoque, A.; Tanzin, A.Z.; Nath, C.; et al. Knowledge, Attitude, and Practices on Antimicrobial Use and Antimicrobial Resistance among Commercial Poultry Farmers in Bangladesh. Antibiotics 2021, 10, 784.

9. Munita, J.M.; Arias, C.A. Mechanisms of antibiotic resistance. Virulence Mechanisms of Bacterial Pathogens, 2016, 481–511.

10. Zhang, L.; Levy, K.; Trueba, G.; Cevallos, W.; Trostle, J.; Foxman, B.; Eisenberg, J.N. Effects of selection pressure and genetic association on the relationship between antibiotic resistance and virulence in Escherichia coli. Antimicrob. Agents Chemother. 2015, 59, 6733–6740.

11. Hassan, M.M.; Amin, K.B.; Ahaduzzaman, M.; Alam, M.; Faruk, M.S.; Uddin, I. Antimicrobial resistance pattern against Escherichia coli and Salmonella in layer poultry. Res. J. Vet. Pract. 2014, 2, 30–35.

12. Van den Bogaard, A.E.; London, N.; Driessen, C.A.G.G.; Stobberingh, E.E. Antibiotic resistance of faecal Escherichia coli in poultry, poultry farmers, and poultry slaughterers. J. Antimicrob. Chemother. 2001, 47, 763–771.

13. McEwen, S.A.; Collignon, P.J. Antimicrobial resistance: a one health perspective. Antimicrob. Resist. Bact. Livest. Companion Anim. 2018, 521–547.

14. Osman, K.M.; Kappell, A.D.; Elhadidy, M.; ElMougy, F.; El-Ghany, W.A.A.; Orabi, A.; Yousef, H.M. Poultry hatcheries as potential reservoirs for antimicrobial-resistant Escherichia coli: A risk to public health and food safety. Sci. Rep. 2018, 8, 5859.

15. Nath, C.; Das, T.; Islam, M.S.; Hasib, F.Y.; Singha, S.; Dutta, A.; Barua, H.; Islam, M.Z. Colistin resistance in multidrug-resistant Escherichia coli isolated from retail broiler meat in Bangladesh. Microb. Drug Resist. 2023, 29, 523–532.

16. Bélanger, L.; Garenaux, A.; Harel, J.; Boulianne, M.; Nadeau, E.; Dozois, C.M. Escherichia coli from animal res-ervoirs as a potential source of human extraintestinal pathogenic E. coli. FEMS Immunol. Med. Microbiol. 2011, 62, 1–10.

17. Islam, A.; Saifuddin, A.; Al Faruq, A.; Islam, S.; Shano, S.; Alam, M.; Hassan, M.M. Antimicrobial residues in tissues and eggs of laying hens at Chittagong, Bangladesh. Int. J. One Health 2016, 2, 75–80.

18. Alam, G.S.; Hassan, M.M.; Ahaduzzaman, M.; Nath, C.; Dutta, P.; Khanom, H.; Cobbold, R. Molecular detection of tetracycline-resistant genes in multidrug-resistant Escherichia coli isolated from broiler meat in Bangladesh. Antibiotics 2023, 12, 418.

19. Tanzin, A.Z.; Nath, C.; Nayem, M.R.; Sayeed, M.A.; Khan, S.A.; Magalhaes, R.S.; Alawneh, J.I.; Hassan, M.M. Detection and Characterisation of Colistin-Resistant Escherichia coli in Broiler Meats. Microorganisms. 2024,12(12):2535.

20. Malorny, B.; Hoorfar, J.; Hugas, M.; Heuvelink, A.; Fach, P.; Ellerbroek, L.; Helmuth, R. Interlaboratory diagnostic accuracy of a Salmonella-specific PCR-based method. Int. J. Food Microbiol. 2003, 89, 241–249.

21. Godambe, L.P.; Bandekar, J.; Shashidhar, R. Species-specific PCR-based detection of Escherichia coli from Indian foods. 3 Biotech 2017, 7, 1–5.

22. Bauer, A.W.; Kirby, W.M.M.; Sherris, J.C.; Turck, M. Antibiotic susceptibility testing by a standardized single disk method. Am. J. Clin. Pathol. 1966, 45, 493–496.

23. CLSI. M100: Performance standards for antimicrobial susceptibility testing. 2020. Available online: https://www.nih.org.pk/wp-content/uploads/2020/02/CLSI-2020.pdf (accessed on 20 September 2024).

24. Magiorakos, A.P.; Srinivasan, A.; Carey, R.B.; Carmeli, Y.; Falagas, M.E.; Giske, C.G.; Monnet, D.L. Multi-drug-resistant, extensively drug-resistant, and pandrug-resistant bacteria: An international expert proposal for interim standard definitions for acquired resistance. Clin. Microbiol. Infect. 2012, 18, 268–281.

25. Krumperman, P.H. Multiple Antibiotic Resistance Indexing of Escherichia coli to Identify High-Risk Sources of Fecal Contamination of Foods. Appl. Environ. Microbiol. 1983, 46(1), 165–170

26. Lanz, R.; Kuhnert, P.; Boerlin, P. Antimicrobial resistance and resistance gene determinants in clinical Escherichia coli from different animal species in Switzerland. Vet. Microbiol. 2003, 91, 73–84.

27. Hasman, H.; Mevius, D.; Veldman, K.; Olesen, I.; Aarestrup, F.M. β-Lactamases among extended-spectrum β-lactamase (ESBL)-resistant Salmonella from poultry, poultry products, and human patients in The Nether-lands. J. Antimicrob. Chemother. 2005, 56, 115–126.

28. Hossain, M.T.; Siddique, M.P.; Hossain, F.M.A.; Zinnah, M.A.; Hossain, M.M.; Alam, M.K.; Choudury, K.A. Isolation, identification, toxin profile, and antibiogram of Escherichia coli isolated from broilers and layers in the Mymensingh district of Bangladesh. Bangl. J. Vet. Med. 2008, 6, 1–5.

29. Jakaria, A.T.M.; Islam, M.A.; Khatun, M.M. Prevalence, characteristics, and antibiogram profiles of Escherichia coli isolated from apparently healthy chickens in Mymensingh, Bangladesh.

30. Bashar, T.; Rahman, M.; Rabbi, F.A.; Noor, R.; Rahman, M.M. Enterotoxin profiling and antibiogram of Escherichia coli isolated from poultry feces in Dhaka district of Bangladesh. Stamford J. Microbiol. 2011, 1, 51–57.

31. Islam, M.J.; Sultana, S.; Das, K.K.; Sharmin, N.; Hasan, M.N. Isolation of plasmid-mediated multidrug-resistant Escherichia coli from poultry. Int. J. Sustain. Crop Prod. 2008, 3, 46–50.

32. Schroeder, C.M.; Meng, J.; Zhao, S.; DebRoy, C.; Torcolini, J.; Zhao, C.; White, D.G. Antimicrobial resistance of Escherichia coli O26, O103, O111, O128, and O145 from animals and humans. Emerg. Infect. Dis. 2002, 8, 1409.

33. Parvin, M.S.; Talukder, S.; Ali, M.Y.; Chowdhury, E.H.; Rahman, M.T.; Islam, M.T. Antimicrobial Resistance Pattern of Escherichia coli Isolated from Frozen Chicken Meat in Bangladesh. Pathogens 2020, 9, 420

34. G. Jacoby, β-Lactamase classification and amino acid sequences for TEM, SHV and OXA extended-spectrum and inhibitor resistant enzymes, http://www lahey org/Studies/ (2012).

35. Li, X.; Watanabe, N.; Xiao, C.; Harter, T.; McCowan, B.; Liu, Y.; Atwill, E.R. Antibiotic-resistant Escherichia coli in surface water and groundwater in dairy operations in Northern California. Environ. Monit. Assess. 2014, 186, 1253–1260.

36. Hasan, B.; Faruque, R.; Drobni, M.; Waldenström, J.; Sadique, A.; Ahmed, K.U.; Alam, M. High prevalence of antibiotic resistance in pathogenic Escherichia coli from large- and small-scale poultry farms in Bangladesh. Avian Dis. 2011, 55, 689–692.

37. Islam, A.; Saifuddin, A.K.M.; Al Faruq, A.; Islam, S.; Shano, S.; Alam, M.; Hassan, M.M. Antimicrobial residues in tissues and eggs of laying hens at Chittagong, Bangladesh. Int. J. One Health 2016, 2, 75–80.

38. Sobur, M.A.; Sabuj, A.A.M.; Sarker, R.; Rahman, A.T.; Kabir, S.L.; Rahman, M.T. Antibiotic-resistant Escherichia coli and Salmonella spp. associated with dairy cattle and farm environment having public health significance. Vet. World 2019, 12, 984.

39. Ferdous, J.; Bradshaw, A.; Islam, S.A.; Zamil, S.; Islam, A.; Ahad, A.; Hoque, M.A. Antimicrobial residues in chicken and fish, Chittagong, Bangladesh. EcoHealth 2019, 16, 429–440.

40. Sattar, S.; Hassan, M.M.; Islam, S.K.M.; Alam, M.; Al Faruk, M.S.; Chowdhury, S.; Saifuddin, A.K.M. Antibiotic residues in broiler and layer meat in Chittagong district of Bangladesh. Vet. World 2014, 7, 738–742.

41. Couper, M.R. Strategies for the rational use of antimicrobials. Clin. Infect. Dis. 1997, 24, S154–S156.

42. Kalam, M.A.; Alim, M.A.; Shano, S.; Nayem, M.R.K.; Badsha, M.R.; Mamun, M.A.A.; Hassan, M.M. Knowledge, attitude, and practices on antimicrobial use and antimicrobial resistance among poultry drug and feed sellers in Bangladesh. Vet. Sci. 2021, 8, 111.

43. Okeke, I.N.; Lamikanra, A.; Edelman, R. Socioeconomic and behavioral factors leading to acquired bacterial resistance to antibiotics in developing countries. Emerg. Infect. Dis. 1999, 5, 18.

44. Barbosa, T.M.; Levy, S.B. The impact of antibiotic use on resistance development and persistence. Drug Resist. Updat. 2000, 3, 303–311.

45. Levy, S.B. The challenge of antibiotic resistance. Sci. Am. 1998, 278, 46–53.

46. Hofacre, C.L.; Fricke, J.A.; Inglis, T. Antimicrobial drug use in poultry. Antimicrob. Ther. Vet. Med. 2013, 569–587.

47. Summers, A.O. Genetic linkage and horizontal gene transfer: The roots of the antibiotic multi-resistance problem. Anim. Biotechnol. 2006, 17, 125–135.

48. Gill, C.O. Microbiological conditions of meats from large game animals and birds. Meat Sci. 2007, 77, 149–160.

98. Gandra, S.; Kotwani, A. Need to improve availability of "access" group antibiotics and reduce the use of "watch" group antibiotics in India for optimum use of antibiotics to contain antimicrobial resistance. J. Pharm. Policy Pract. 2019, 12, 1–4.

50. Hsia, Y.; Lee, B.R.; Versporten, A.; Yang, Y.; Bielicki, J.; Jackson, C.; Dedeic-Ljubovic, A. Use of the WHO Access, Watch, and Reserve classification to define patterns of hospital antibiotic use (AWaRe): An analysis of pediatric survey data from 56 countries. Lancet Glob. Health 2019, 7, e861–e871.

51. Barnett, M.L. The Rationale for the Daily Use of an Antimicrobial Mouthrinse. J. Am. Dent. Assoc. 2006, 137, S16–S21.

52. Moellering, R.C., Jr. Rationale for Use of Antimicrobial Combinations. Am. J. Med. 1983, 75, 4–8.

Click here to access/download
Supporting Information
Supplementary Table S1.docx
